# Evaluating the Accuracy of Chest CT in Detecting COVID-19 Through Tracheobronchial Wall Thickness: Insights From Emergency Department Patients in Mid-2023

**DOI:** 10.7759/cureus.69161

**Published:** 2024-09-11

**Authors:** Akiyuki Kotoku, Hiroki Horinouchi, Tatsuya Nishii, Midori Fukuyama, Yasutoshi Ohta, Tetsuya Fukuda

**Affiliations:** 1 Radiology, National Cerebral and Cardiovascular Center, Suita, JPN

**Keywords:** covid-19, diagnostic imaging, sars-cov-2, tomography, trachea, x-ray computed

## Abstract

Background

The post-pandemic phase of the coronavirus infectious disease that emerged in 2019 (COVID-19) has necessitated updates in radiology, with emerging evidence suggesting tracheobronchial wall thickness as a potential new diagnostic marker.

Purpose

To evaluate the accuracy of chest computed tomography (CT) scans in identifying COVID-19 by assessing tracheobronchial wall thickness in mid-2023.

Material and methods

A retrospective review was conducted on 60 patients who underwent thoracoabdominal CT and the severe acute respiratory syndrome coronavirus (SARS-CoV-2) antigen tests during emergency visits between June and August 2023. Tracheobronchial wall thickness was measured using a 4-point scale (1=no thickening, 2=mild, 3=moderate, 4=significant). Lung assessment employed the COVID-19 Reporting and Data System (CO-RADS). Patients were classified based on antigen test results. The Mann-Whitney U test and Fisher's exact test compared characteristics and CT findings. Diagnostic performance was evaluated using the area under the receiver operating characteristic curves (AUC).

Results

The SARS-CoV-2-positive group showed significantly thicker tracheobronchial walls (median 1.5 mm vs. 1.2 mm, P < 0.001), especially in the trachea's membranous wall (median 1.2 mm vs. 0.9 mm, P < 0.001) and higher scores (median 3 vs. 2, P < 0.001). CO-RADS scores showed no significant difference. Quantitative and qualitative wall thickness assessments demonstrated high diagnostic value, with AUCs of 0.90 and 0.94, and accuracies of 85% and 87%, respectively.

Conclusion

Tracheobronchial wall thickness on chest CT exhibited high diagnostic accuracy, establishing it as a reliable marker for COVID-19 detection in mid-2023.

## Introduction

The medical landscape has experienced substantial transformations since the onset of the severe acute respiratory syndrome coronavirus (SARS-CoV-2) [1]. Currently, we are in the post-pandemic phase, marked by viral mutations, treatment advancements, and vaccine development. The radiology department has also experienced considerable changes from its previous routine. Yet, it remains imperative for radiologists to stay updated on the imaging aspects of the coronavirus infectious disease that emerged in 2019 (COVID-19). In the initial stages of the pandemic, chest computed tomography (CT) scans were employed to identify distinct ground-glass opacities as crucial indicators of COVID-19 [2,3]. These CT findings triggered extensive global research on the accuracy of the disease diagnosis [4-7] and their prognostic implications [8]. Chest CT has evolved into a pivotal diagnostic tool for COVID-19, enabling early detection before the polymerase chain reaction (PCR) testing, particularly in situations where PCR testing is constrained [9]. Moreover, research has validated the high diagnostic accuracy of CT, not only for detecting the disease but also for evaluating its severity [10] and predicting its progression [11]. Thus, beyond its significance in COVID-19 cases, radiology departments have swiftly adapted during the pandemic [12].

In comparison to the earlier phases of the pandemic, there has been an increase in the number of vaccinated patients, accompanied by a decrease in the typical pneumonia presentation [8,13]. The emergence of the Omicron (B.1.1.529) variant has influenced clinical manifestations and disease progressions, reducing the prevalence of typical pneumonia presentations [13-15]. These shifts prompt a reconsideration of the relevance of pre-Omicron chest CT imaging findings in current diagnostic strategies. In 2023, one case report [16] and our initial observations indicated an elevation in tracheal and central bronchial wall thickness in SARS-CoV-2-positive cases. This observation may hold significance for the CT-based diagnosis of COVID-19 and warrants further investigation to validate its diagnostic accuracy. It is important to note that chest CT is no longer recommended as the primary diagnostic tool for assessing SARS-CoV-2 infection [17,18]. However, the incidental detection of COVID-19 cases in patients receiving CT scans in emergency departments is still vital for infection control and ensuring the seamless continuity of hospital operations. For radiologists and healthcare professionals operating in the emergency department, recognizing incidental COVID-19 CT indicators is critical to protecting healthcare workers from infection risks [19]. Therefore, our study aims to determine the diagnostic efficacy of chest CT in detecting COVID-19, with a specific focus on tracheobronchial wall thickness, considering data from mid-2023 onward.

## Materials and methods

Ethics

This retrospective study was approved by our review board (approval number: R19039-3). The requirement for informed consent was waived due to the study design.

Study population

A retrospective analysis was conducted on 162 patients who underwent chest or thoracoabdominal CT during their emergency department visits between June and August 2023. Our hospital specializes in treating cardiovascular and cerebrovascular diseases, with the emergency room primarily for acute cardiovascular and stroke patients. Clinical scenarios for body CT include evaluation of aortic disease and pulmonary embolism from chest and back pain, as well as evaluation of infection focus for associated fever in patients with heart failure or stroke. Out of this cohort, 64 individuals who had undergone SARS-CoV-2 antigen testing were included in the study. During this period, our facility's infection control team established the role of performing antigen testing for patients in the emergency room. Patients with fever or respiratory symptoms, for whom COVID-19 could not be clinically ruled out, were first tested for the antigen in the emergency room. Exclusion criteria comprised patients exhibiting a temporal gap exceeding one day between the administration of the antigen test and the CT examination. Patient characteristics and blood test results were systemically examined utilizing an electronic medical recording system.

Image acquisition and reconstruction

Three distinct CT scanners were employed for conducting CT scans, comprising a 320-area detector CT scanner (Aquilion ONE, Canon Medical System, Otawara, Japan), a 256-area detector CT scanner (Revolution CT, GE Healthcare, IL, USA), and a 192-row dual-source CT scanner (SOMATOM Force, Siemens Healthcare, Forchheim, Germany). Image reconstruction utilized a slice thickness and increment of 5 mm. Further, a thickness and increment of 1 mm or 1.25 mm were employed for the thin-slice data. Detailed imaging and reconstruction parameters are outlined in Table 4 (Appendices).

Image analysis

**Figure 1:**
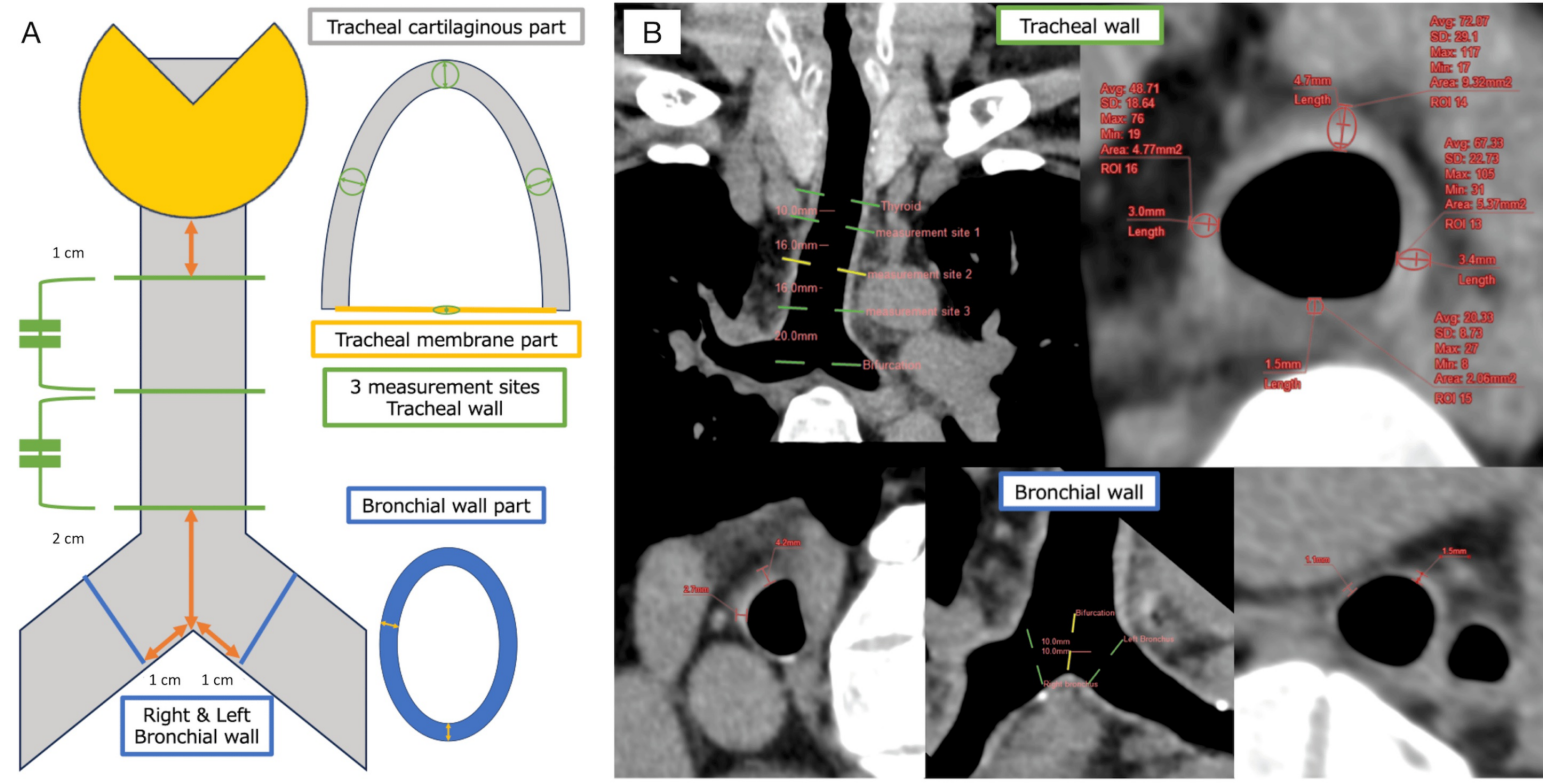
Details of measurements of the tracheobronchial wall thickness The left panel (A) has illustrations for the trachea and main bronchial measurement sites, while the right panel (B) shows actual measurements for tracheal and bronchial wall thicknesses. Tracheal thickness is evaluated at three points and in cross-section at four quadrants to differentiate between membranous and cartilaginous walls. Bronchial thickness is averaged from four measurements in each bronchus.

Board-certified radiologists (A.K., H.H., and T.N, with respective 10, 11, and 15 years of experience) conducted image analysis at a dedicated workstation. The window width and level for tracheobronchial assessment were standardized to 300 and 30, respectively. Quantitative measurements of the tracheobronchial wall were initially performed by a radiologist (A.K.) using thin-slice thickness data (Figure 1).

**Figure 2:**
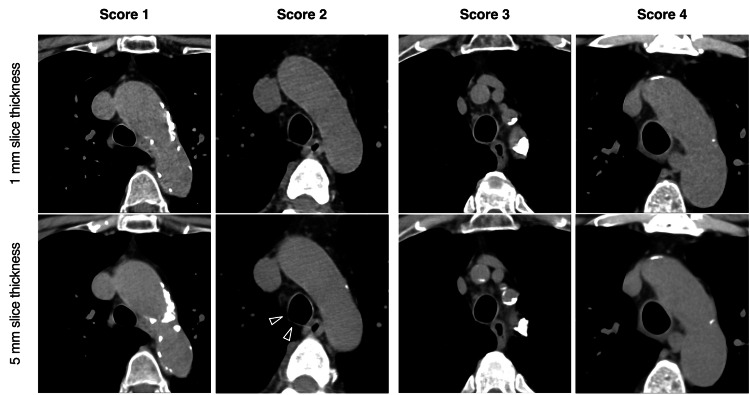
Four-point scoring of tracheobronchial wall thickness Radiologists assessed the thickness of tracheal bronchus walls using both 5 mm and thin slice-thickness data using the 4-point score for thickness (1 indicating no thickening, 2 showing mild thickening not considered pathological, 3 indicating moderate thickening, and 4 indicating significant thickening). Score 2 was determined from score 3 when the tracheal wall could not be followed circumferentially on a 5 mm slice-thick image (black arrowhead).

The tracheobronchial lumen centerline was delineated, and a short-axis image perpendicular to this centerline was reformatted. This facilitated the measurement of the tracheal wall at three specific points: 1 cm below the inferior end of the thyroid gland, 2 cm above the tracheal bifurcation, and at the midpoint between these two locations. The bronchial wall was examined 1 cm distal to the tracheal bifurcation. Tracheal wall thickness and CT values were evaluated on short-axis images at the 0, 3, 6, and 9 o'clock positions. The circular region of interest for CT value measurement was defined using the measured wall thickness as its diameter. Bronchial wall thickness was measured at two designated sites. The 6 o'clock position was considered the membranous wall, the combined 0, 3, and 9 o'clock positions constituted the cartilaginous wall, and the overall average represented the tracheal wall CT values and the thickness. Bronchial wall thickness was defined as the combined average of four measurements in the left and right bronchi. Additionally, radiologists (H.H. and T.N.) assessed the thickness of tracheal and main bronchus walls using both 5 mm and thin slice-thickness data, assessed a 4-point score for thickness (1 indicating no thickening, 2 indicating mild thickening not considered pathological, 3 indicating moderate thickening, and 4 indicating significant thickening) (Figure 2).

In case of discrepant scores, the radiologists collaborated to reach a consensus. The window width and level were set for lung assessment to 1600 and -600, respectively. Board-certified radiologists (A.K. and T.N.) performed the COVID-19 Reporting and Data System (CO-RADS) grading [20] for COVID-19-related findings through consensus reading using thin slice-thickness data. Furthermore, using thin slice-thickness data, pulmonary density scores were evaluated through a deep-learning-based automated measurement system for Chest CT (AI-RAD Companion Chest CT, Siemens). Further, we performed both qualitative and quantitative evaluations of tracheobronchial wall thickening on SARS-CoV-2 positive patients who underwent follow-up CT scans within six months after disease resolution.

SARS-CoV-2 antigen test

The nasopharyngeal swab fluid for quantitative analysis was subjected to rapid quantitative antigen testing using Lumipulse G1200 Plus (Fuji Rebio, Tokyo, Japan) [21], performed by a physician. Physicians selected patients for antigen testing based on the hospital's infection control policies, as previously mentioned. Our hospital classified cases as positive during this period if the antigen count exceeded 10 in the quantitative antigen test. The PCR test was performed for patients with inconclusive antigen test results. The results were retrieved from the electronic medical records.

Statistical analysis

Continuous variables were presented as medians with the interquartile ranges (IQR). All statistical analyses were conducted using JMP Pro 15 (SAS Institute, Cary, NC, USA) and R open-source software (version 4.3.1, https://www.r-project.org/; The R Foundation for Statistical Computing, Vienna, Austria). A significance level of P < 0.05 was applied for all analyses.

Patients were classified based on the detection of COVID-19 confirmed by antigen testing. Univariate analysis employed Mann-Whitney U and Fisher's exact tests to compare patient characteristics and CT findings between the two groups. Statistical power was assessed to account for beta errors arising from sample size effects. Multivariate logistic regression analysis was performed to distinguish these groups based on patient characteristics and CT findings, with factor selection carried out through a stepwise method. The diagnostic performance and net clinical benefit were appraised using the area under the receiver operating characteristic curves (AUCs) derived from the CT findings and decision curve analysis [22]. The differences in the tracheobronchial wall thickening between initial and follow-up CT scans were evaluated using the paired t-test and the Wilcoxon signed-rank test. Cohen's kappa values were calculated to gauge the consistency of inter-observer scoring in CT imaging. Interpretation of the coefficient was as follows: values ≥ 0.81 indicated excellent agreement, 0.80-0.61 substantial, 0.60-0.41 moderate, 0.40-0.21 fair, and ≤ 0.20 poor agreement.

## Results

**Figure 3:**
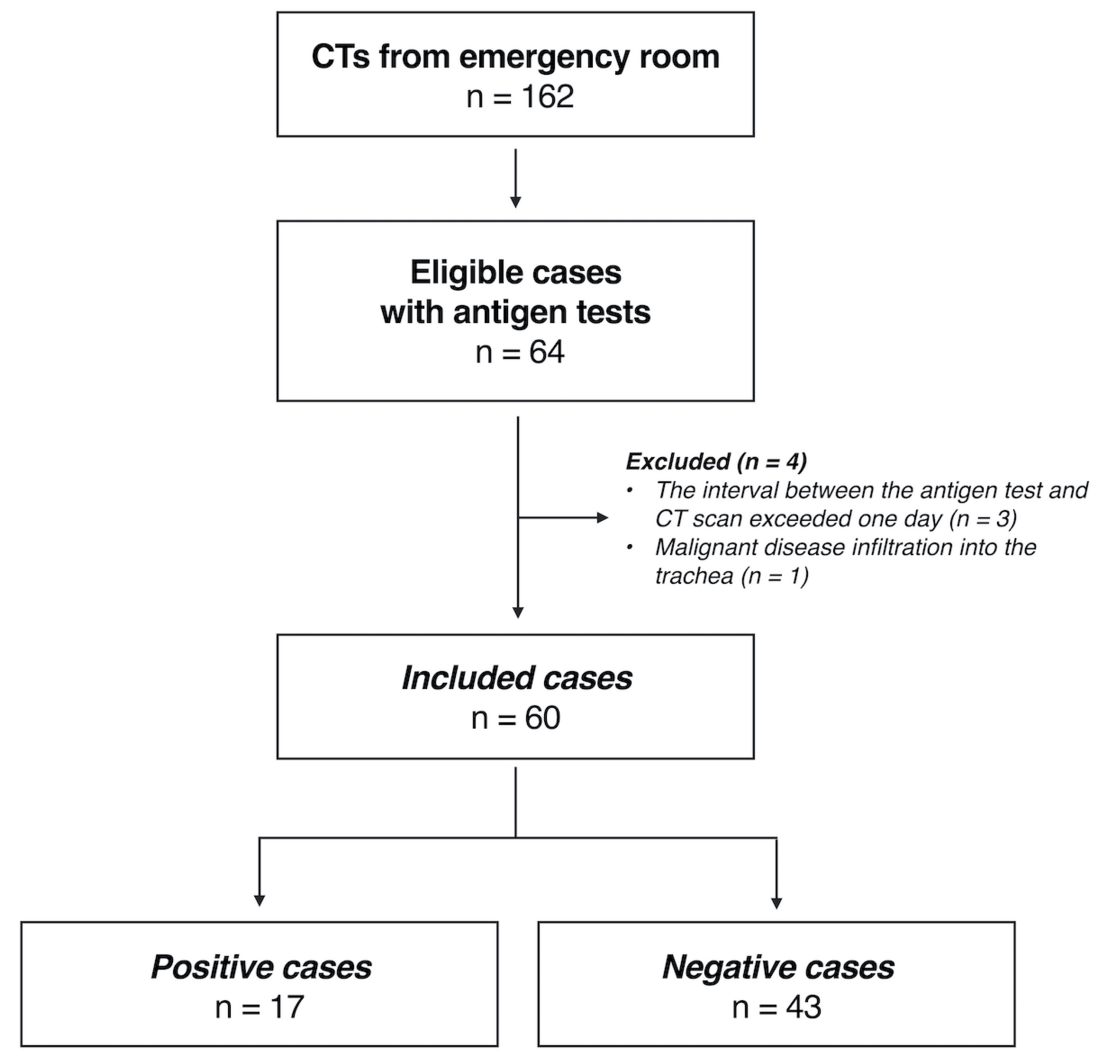
Flowchart of the subjects in this study

CT and antigen testing were conducted on 64 patients, with three cases excluded due to a time difference exceeding one day between CT and antigen tests and one case suspected of esophageal cancer invasion into the trachea. Consequently, 60 patients were included in the study (Figure 3).

All participants were of Asian descent; 37 were male, with a median age of 78 years (IQR 61-86) (Table 1). The patient presented with fever in 39 cases, dyspnea in 37 cases, cough in 14, sore throat in 11, and chest pain in three. The median interval from symptom onset to the CT scan was one day (IQR 1-3 days). The volume-CT dose index was median 9.0 (IQR 7.2-10.2) mGy. The study encompassed three endotracheally intubated patients and two individuals on extracorporeal membrane oxygenation. In terms of CO-RADS grading, the distribution was as follows: grade 0 in one case (2%), grade 1 in 36 subjects (62%), grade 2 in 18 patients (30%), grade 3 in four cases (7%), and grade 5 in one case (2%). Of the 60 patients, 17 tested positive for SARS-CoV-2 antigens, while 43 tested negative. Patient background details are detailed in Table 1. The group testing positive for SARS-CoV-2 was significantly younger, with a higher incidence of sore throat and chest pain than the SARS-CoV-2 negative group. This group also experienced a more extended time between symptom onset and the CT scan, and they exhibited lower white blood cell and platelet counts in blood tests. The negative group comprised 19% of patients (8/43) with bacterial pneumonia and two individuals testing positive for influenza. No significant differences in CO-RADS or pulmonary density scores were observed between the two groups.

**Table 1:** Patients demographics Note: Except where indicated, data are numbers of patients, with percentages in parentheses. † Data are medians, with interquartile ranges in parentheses. CT: computed tomography; CO-RADS: COVID-19 Reporting and Data System

Characteristics	Overall (n = 60)	Negative Group (n = 43)	Positive Group (n = 17)	P-value
Age (y) †	78 (64, 85)	80 (75, 87)	68 (49, 81)	0.02
Male patients	37 (62)	26 (61)	11 (65)	0.99
Body mass index (kg/m^2^) †	20.9 (18.7, 24.5)	20.3 (18.8, 24.5)	21.3 (18.5, 23.8)	0.91
Body temperature (℃) †	37.8 (37.1, 38.6)	37.8 (37.1, 38.9)	37.6 (37.1, 38.1)	0.25
Smoking Status				0.69
Current	7 (12)	5 (12)	2 (12)	
Former	21 (35)	13 (30)	8 (47)	
Never	23 (38)	18 (42)	5 (29)	
Symptoms				
Fever	39 (65)	29 (67)	10 (59)	0.56
Sore throat	11 (18)	3 (7)	8 (47)	0.001
Cough	14 (23)	7 (16)	7 (41)	0.09
Short of breath	37 (62)	27 (63)	10 (59)	0.78
Chest pain	3 (5)	0 (0)	3 (18)	0.02
Onset to CT interval (day) †	1 (1, 3)	1 (1, 2)	2 (1, 5)	0.01
Blood test				
C-reactive protein (mg/dL) †	3.62 (0.87, 9.44)	5.21 (0.92, 9.77)	3.15 (0.87, 6.07)	0.47
White blood count (×10^3^/μL) †	9.4 (6.7, 11.7)	10.2 (7.9, 14.0)	7.1 (6.0, 8.2)	<0.001
Platelet count (×10^3^/μL) †	181.0 (140.8, 250.8)	223.0 (154.5, 263.0)	153.0 (104.0, 177.0)	0.004
Influenza antigen test				0.08
Negative	36 (60)	29 (67)	7 (42)	
Positive	2 (3)	2 (5)	0 (0)	
No test	22 (37)	12 (28)	10 (59)	
Treatments				
Intubation	4 (7)	3 (7)	1 (6)	1.0
Extracorporeal membrane oxygenation	2 (3)	0 (0)	2 (12)	0.08
CT findings				
CO-RADS grading				0.22
0	1 (2)	1 (2)	0 (0)	
1	36 (60)	25 (58)	11 (65)	
2	18 (30)	15 (35)	3 (18)	
3	4 (7)	2 (5)	2 (12)	
5	1 (2)	0 (0)	1 (6)	
Lung density score †	3 (2, 5)	3 (2, 5)	3 (1, 5)	0.32

**Figure 4:**
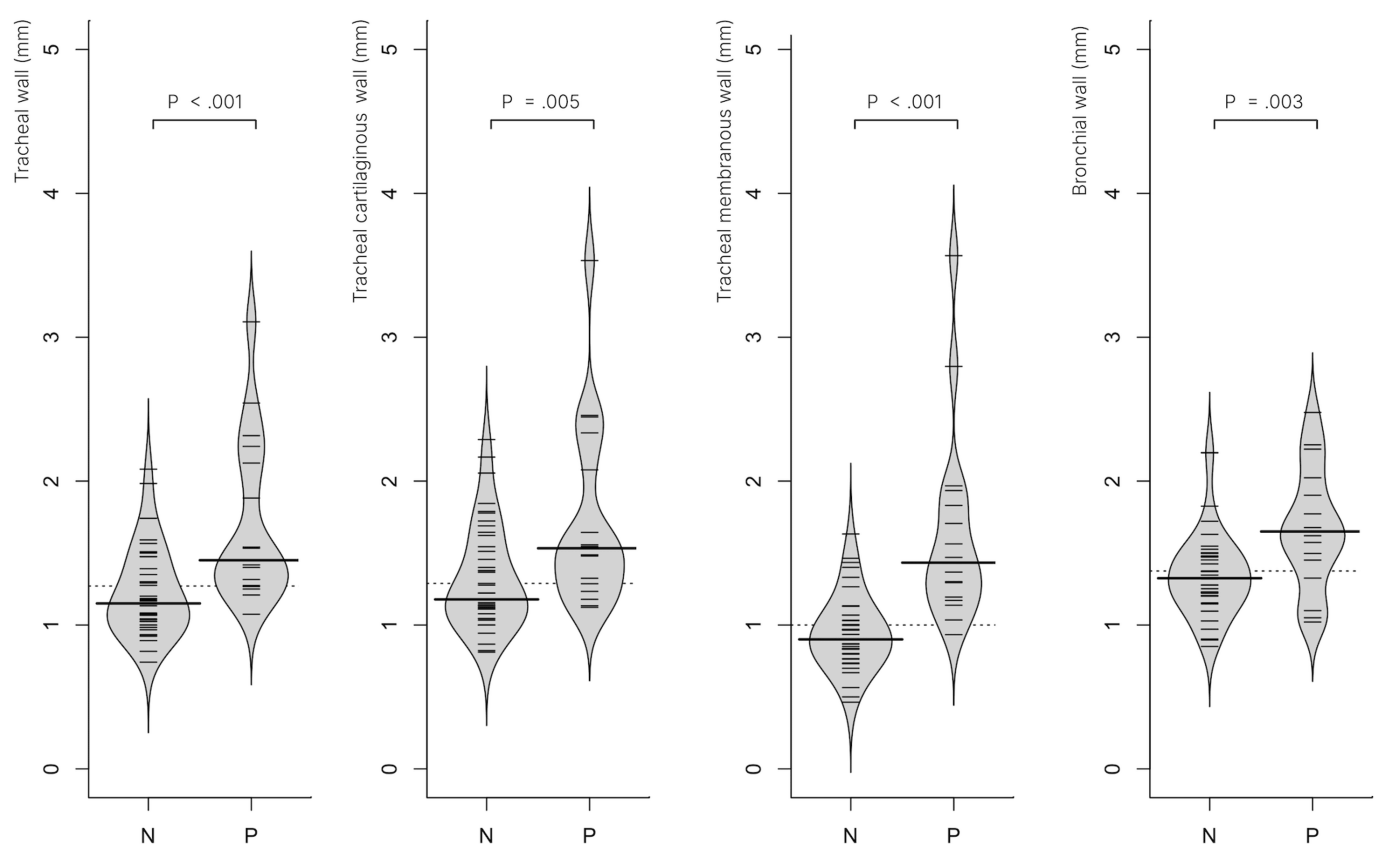
Bean plots of the results of measurements of the tracheobronchial wall thickness Note: N: SARS-CoV-2 antigen negative group; P: SARS-CoV-2 antigen positive group

The median measurements for tracheal wall thickness, the membranous wall, cartilaginous wall, and bronchi were 1.3 mm (IQR, 1.0-1.5), 1.0 mm (IQR, 0.8-1.3), 1.3 mm (IQR, 1.1-1.6), and 1.4 mm (IQR, 1.2-1.6), respectively. The positive group exhibited significantly greater thickness in tracheal membranous walls (P < 0.001, power = 0.99), cartilaginous walls (P < 0.005, power = 0.87), tracheal walls (P < 0.001, power = 0.99), and bronchial wall (P < 0.003, power = 0.92) compared to the negative group. The results are illustrated in Figure 4 and summarized in Table 2.

**Table 2:** Thickness and CT values for the tracheal and bronchial wall Note: Data are medians, with interquartile ranges in parentheses.

	Overall	Negative Group	Positive Group	P-value
Quantitative Analysis				
Wall thickness (mm)				
Tracheal wall	1.3 (1.0, 1.5)	1.2 (1.0, 1.4)	1.5 (1.3, 2.1)	<0.001
Cartilaginous wall	1.3 (1.1, 1.6)	1.2 (1.1, 1.5)	1.5 (1.3, 2.1)	0.005
Membranous wall	1.0 (0.8, 1.3)	0.9 (0.8, 1.1)	1.4 (1.2, 1.8)	<0.001
Bronchial wall	1.4 (1.2, 1.6)	1.3 (1.2, 1.5)	1.7 (1.5, 1.9)	0.003
Wall density (HU)				
Tracheal wall	27 (18, 41)	24 (16, 38)	36 (26, 45)	0.10
Cartilaginous wall	32 (20, 52)	29 (19, 50)	39 (30, 56)	0.29
Membranous wall	15 (1, 26)	13 (-6, 22)	26 (10, 32)	0.02
Qualitative Analysis				
Score	2 (1, 3)	2 (1, 2)	4 (3, 4)	<0.001

CT values for the tracheal membranous wall were higher in the positive group than in the negative group (Table 2).

Similarly, in the assessment of tracheobronchial thickening by radiologists, the positive group demonstrated a significantly higher score (median 3, IQR 3-4 vs. 2, IQR 1-2, P < 0.001, power = 0.99). Multivariate analysis identified tracheal membranous thickening, as determined by the CT, as an independent factor (adjusted odds ratio, 257, 95% confidence interval (CI): 15.9-14761, P = 0.001), along with white blood cell counts (adjusted odds ratio, 0.62, 95% CI: 0.38-0.86, P = 0.02).

**Figure 5:**
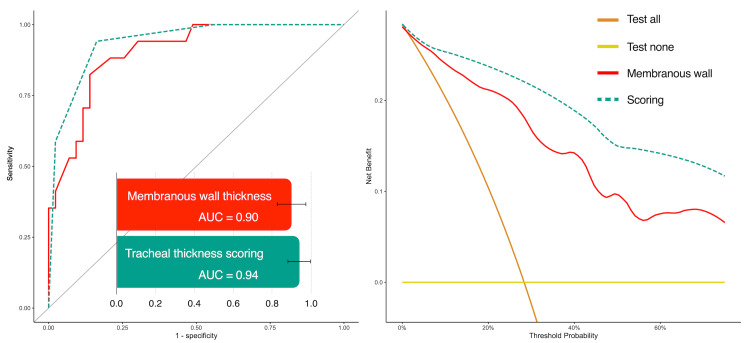
Receiver operating characteristic curves and decision curves Both quantitative evaluation of tracheal membranous wall thickening and qualitative evaluation of tracheal wall thickening had high accuracy in the receiver operating characteristic curve analysis, achieving the area under the curve (AUC) values of 0.90 and 0.94, respectively. Decision curve analysis showed high net benefits across all pretest probability thresholds, indicating the effectiveness of both methods in diagnosing tracheal wall thickening.

The receiver operating characteristic (ROC) analysis demonstrated a sensitivity of 88%, a specificity of 84%, and an accuracy of 85% when utilizing a cutoff of 1.2 mm for tracheal membranous thickness. The corresponding AUC was 0.90 (95% CI: 0.83-0.98) (Figure 5 and Table 3).

**Table 3:** Diagnostic performances Note: Numbers in parentheses are raw data and 95% confidence intervals. † P-values of the DeLong test were 0.02 for vs. Tracheal wall, 0.004 for vs. Cartilaginous wall, and 0.08 for vs. Bronchial wall.

	Tracheal Wall	Cartilaginous Wall	Membranous Wall	Bronchial Wall	Tracheal Thickness Scoring
Cutoff (mm)	1.2 (1.2, 1.5)	1.4 (1.2, 1.5)	1.2 (1.0, 1.3)	1.5 (1.4, 1.6)	Score ≥3
Sensitivity (%)	88 (15/17) (65, 100)	77 (13/17) (53, 94)	88 (15/17) (71, 100)	71 (12/17) (53, 88)	94 (16/17) (77, 100)
Specificity (%)	67 (29/43) (51, 86)	67 (29/43) (49, 84)	84 (36/43) (72, 95)	84 (36/43) (63, 95)	84 (36/43) (72, 98)
Accuracy (%)	73 (44/60) (62, 83)	70 (42/60) (57, 80)	85 (51/60) (75, 93)	80 (48/60) (63, 90)	87 [52/60) (78, 95)
Area under the curve	0.79 (0.68, 0.91)	0.73 (0.60, 0.87)	0.90 ^†^ (0.83, 0.98)	0.75 (0.59, 0.91)	0.94 (0.88, 0.99)

Additionally, tracheobronchial thickening scoring (score ≥ 3) exhibited a sensitivity of 94%, a specificity of 84%, and an accuracy of 87% with an AUC of 0.94 (95%CI: 0.88-0.99). The decision curve analyses revealed a high clinical net benefit for both membranous thickness and scoring across all pretest probabilities. The interobserver reproducibility of scores was excellent (kappa = 0.84, 95% CI: 0.73-0.95).

**Figure 6:**
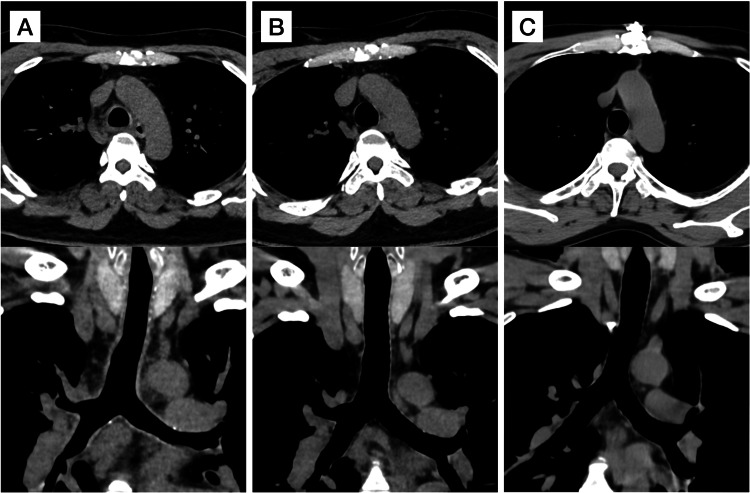
Representative cases Chest CT axial (upper row) and coronal (lower row) images from individual cases are presented. The images compare those of a 48-year-old man diagnosed with COVID-19 at admission (A) with images from one year prior (B), highlighting a circumferential and diffuse thickening of the trachea and main bronchus wall. In contrast, chest CT images of a 50-year-old man from the negative group (C) exhibit no tracheobronchial wall thickening.

**Figure 7:**
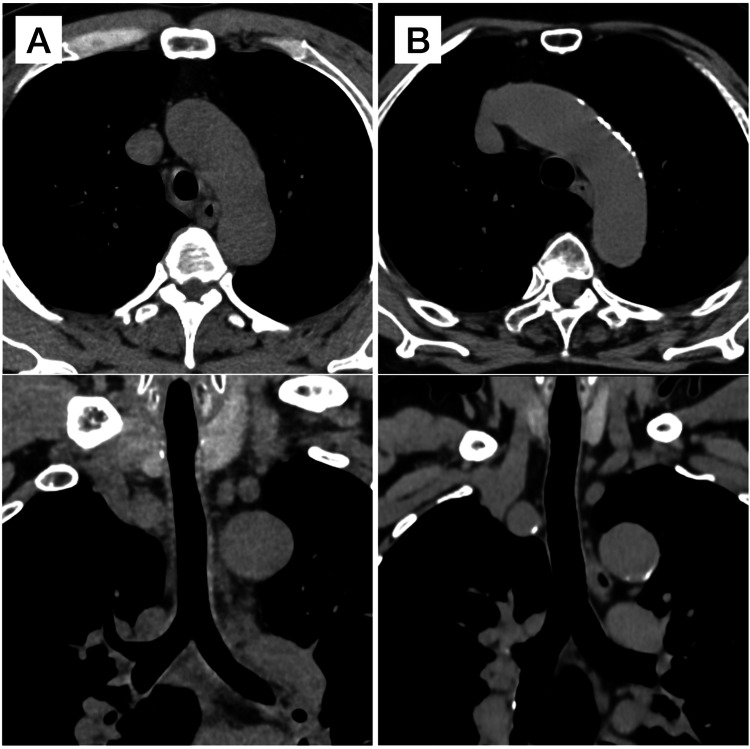
Chest CT images in influenza-positive cases This figure shows chest computed tomography (CT) images in axial (upper row) and coronal (lower row) views. The patients in the images tested positive for influenza but negative for COVID-19. The images of a 56-year-old man (A) display a diffuse thickening of the tracheal and bronchial walls, similar to the thickening observed in COVID-19 cases. In contrast, the images from an 84-year-old woman (B) show no such abnormalities and are akin to those observed in normal cases.

In the four patients who tested positive for SARS-CoV-2 and underwent follow-up CT (median follow-up: 62 days), the average reduction in thickness from the initial to the follow-up CT was -0.5 mm (95% CI: -0.9 to -0.2) for the tracheobronchial wall and -0.7 mm (95% CI: -1.3 to -0.1) for the membranous wall (Table 5, Appendices). The tracheobronchial wall thickening score decreased from an initial median of 3 (IQR, 2-4) to 1 (IQR, 1-2) at follow-up (P = 0.13). In Figure 6A, tracheobronchial thickening is illustrated in the SARS-CoV-2 positive group, while Figure 6B compares it with a previous CT scan. Figure 6C provides a contrast with the SARS-CoV-2 negative group. Figure 7 (Appendices) presents two cases from the SARS-CoV-2 negative group, both positive for the influenza virus, with one exhibiting significant tracheal wall thickening and the other without.

## Discussion

In this study, chest CT scans of patients in an emergency room setting were analyzed, with a focus on tracheobronchial wall thickness in SARS-CoV-2-positive individuals. The SARS-CoV-2-positive group exhibited a significantly thicker tracheobronchial wall, with the membranous wall of the trachea identified as an independent factor in the multivariate analysis. As indicated by the AUC, diagnostic performance was notably high, reaching 0.90 and 0.94 for quantitative and qualitative radiologist evaluations, respectively. No significant differences were observed in CO-RADs or pulmonary density scores between the groups.

The SARS-CoV-2-positive group demonstrated marked diffuse thickening of the tracheobronchial wall, discernible both quantitatively and qualitatively. Further, subjects who underwent follow-up CT scans showed significant decreases in tracheobronchial wall thickening, suggesting that the thickening could be attributed to SARS-CoV-2 infection. Despite the typically thin nature of the membranous part, the observed thickening remained a significant independent factor in multivariate analysis, even when clinical information was considered. The four-point qualitative evaluation by radiologists proved highly diagnostic and reproducible, making it a valuable tool for daily CT image interpretation. However, diffuse thickening of the tracheobronchial wall is not unique to COVID-19 and may also occur in conditions such as amyloidosis and tracheal tuberculosis [23-26]. In this study, a similar incidental finding was noted in one case that tested positive for influenza. Additionally, age-related changes are known to contribute to tracheal wall thickening. Thus, when previous imaging is available, it is essential to reference these to assess the pathological wall thickening. Moreover, close collaboration between radiologists and clinicians is necessary to correlate tracheobronchial findings with clinical data, facilitating a comprehensive imaging diagnosis.

Our study aligns with prior research on SARS-CoV-2, particularly the Omicron variant, highlighting its affinity for angiotensin-converting enzyme-2 receptors in the trachea and bronchi [27,28]. SARS-CoV-2 infection has been reported to induce upper respiratory tract inflammation [29], and tracheobronchial thickening observed in our study confirms these pathophysiological findings. The recent report of the prostate-specific membrane antigen positron emission tomography accumulation in tracheal wall thickening in patients with COVID-19 [30] indicated that this CT imaging finding may reflect inflammation-induced changes [14]. Conversely, the lack of significant differences in pulmonary findings, such as CO-RADs, questions the sole reliance on characteristic pneumonia for COVID-19 diagnosis by 2023. These results presumably reflect changes in imaging findings due to vaccination [8,13] and viral mutations [14,15] since the beginning of the pandemic. The radiological presentation of SARS-CoV-2 has changed due to rapid mutations, necessitating adaption to varying imaging characteristics, including evaluation of CT findings, to ensure consistency with future variants [15].

This study has limitations that need to be addressed. Firstly, it was a single-center retrospective study, potentially subject to selection bias due to the emergency room physicians' discretionary use of antigen tests and chest CT scans. It is also important to note that the pretest probability of COVID-19 infection is relatively low because our hospital is a dedicated cardiovascular and cerebrovascular center, not a center for infectious diseases. Secondly, reliance on antigen tests and the high prevalence of COVID-19 during the study period may have affected diagnostic accuracy. Generalization of these findings should be approached cautiously, considering these factors. While the influence of the small sample size cannot be ruled out, the quantitative and qualitative tracheal wall thickening results were statistically valid, with a statistical power exceeding 0.90. Thirdly, our study did not explore the genetic polymorphism of the virus. However, the predominant strain in Japan during this period was the Omicron variant (XBB.1.16, EG.5, and XBB.1.9.1; top 3, accounting for 60% of the total). Additionally, there are limitations to the possibility of viral coinfections in the COVID-19 cohort and the focus on Omicron variants without analyzing pre-Omicron variants.

## Conclusions

In conclusion, tracheobronchial wall thickness observed on chest CT was a significant marker for detecting COVID-19 in 2023, demonstrating high diagnostic accuracy. Notably, the thickening of the membranous portion of the trachea is particularly distinct and valuable for interpretation. By assessing these tracheal findings, radiologists can provide crucial information to protect not only the patient but also emergency room staff from potential infection, extending beyond the typical lung area findings. However, it is important to note that similar tracheal wall thickening can occur in other viral infections, underscoring the necessity of a comprehensive diagnostic approach.

## Appendices

**Table 4:** Scan and reconstruction parameters * Two distinct reconstruction methodologies were applied: deep learning reconstruction using AiCE Body sharp (Strength Level Standard, Canon Medical System) and TrueFidelity (Strength Level High, GE Healthcare) or medium kernel with iterative reconstruction using Bv40 with ADMIRE (Strength Level 3, Siemens). NI: noise index; SD: standard deviation

Parameters	SOMATOM Force		Revolution CT		Aquilion ONE	
Scanning mode	High-speed scan	Normal scan	High-speed scan	Normal scan	High-speed scan	Normal scan
	Turbo-flash scan	Single-helical scan	Hyperdrive scan	Helical scan	Variable pitch scan	Helical scan
Scan parameters						
Tube voltage (kV)	120	120	120	120	120	120
Automatic tube current setting	CARE Dose4D (Quality ref. mAs,180)	CARE Dose4D (Quality ref. mAs,180)	Organ Dose Modulation (5mm with NI 8)	Organ Dose Modulation (5mm with NI 8)	Volume EC (5mm with SD 8)	Volume EC (5mm with SD 8)
Collimation (mm)	2×192×0.6	96×0.6	125×0.625	125×0.625	160×0.5	160×0.6
Rotation speed (ms)	250	500	280	500	275	500
Pitch	2.4	0.8	1.531	0.995	1.188 for the chest, 0.813 for the abdomen	0.637
Reconstruction parameters*						
Field of view (mm)	400	400	400	400	400	400
Kernel	Bv40	Bv40	Standard	Standard	Body sharp	Body sharp
Strength	ADMIRE 3	ADMIRE 3	TrueFidelity High	TrueFidelity High	AiCE Standard	AiCE Standard

**Table 5:** The difference in tracheobronchial wall thickness from the initial to the follow-up CT in the SARS-CoV-2 positive group Note: Data in parentheses are 95% confidence intervals. SARS-CoV-2: severe acute respiratory syndrome coronavirus; CT: computed tomography

	Mean Difference, Initial to Follow-Up CT	P-value
Quantitative wall thickness (mm)		
Tracheal wall	-0.5 (-0.9, -0.2)	0.02
Cartilaginous wall	-0.6 (-1.2, 0.1)	0.08
Membranous wall	-0.7 (-1.3, -0.1)	0.03
Bronchial wall	-1.0 (-2.0, 0.1)	0.06
